# Massage in Recent Fractures

**Published:** 1900-06-09

**Authors:** 


### MASSAGE IN RECENT FRACTURES.

Speaking at St. George's Hospital a little time ago on the use of massage in the treatment of recent fractures, Mr. William Bennett described the various movements and forms of massage which could be practised in the early stages of fractures, dislocations, and sprains and bruises. He laid special stress upon the effect of smooth-stroking massage in relieving the muscular spasm which so greatly complicates many cases of fracture, especially those of the leg bones. The manœuvre may be described as a smooth, upward movement of the hand, which grasps as much of the circumference of the limb as possible. Not only does this relieve spasm, but it helps to bring about absorption of the effused blood with extreme rapidity. Later on, that is after a few days, some passive movement may be combined with the smooth massage, and then various other forms of massage may be employed, but the great rule is that whatever manœuvres may be necessary later, they must always be preceded by smooth massage, which soothes the irritable muscles so completely that movements of the complete kind can be carried out without exciting muscular contraction of a harmful kind. Mr. Bennett seems to have found that the practical applicability of massage as a routine treatment in cases of fracture is apt to be somewhat restricted by the difficulty of getting it done, and we can have but little doubt that a general practitioner will be well advised if he does for himself such massage as he may think necessary. Mr. Bennett has done well in explaining the whole subject to his students, for so long as a surgeon is responsible for the progress of his case he will do well to be very cautious about handing over to anyone else a detail in the treatment which, while so effectual for good if done with care, might easily come to be saddled with the blame for any evil consequences which the accident might leave behind.

